# An *Arabidopsis* Clathrin Assembly Protein with a Predicted Role in Plant Defense Can Function as an Adenylate Cyclase

**DOI:** 10.3390/biom8020015

**Published:** 2018-03-23

**Authors:** Patience Chatukuta, Tshegofatso B. Dikobe, David T. Kawadza, Katlego S. Sehlabane, Mutsa M. Takundwa, Aloysius Wong, Chris Gehring, Oziniel Ruzvidzo

**Affiliations:** 1 Department of Botany, School of Biological Sciences, North-West University, Private Bag X2046, Mmabatho 2735, South Africa; patience.chatukuta@gmail.com (P.C.); Tshegofatso.Dikobe@nwu.ac.za (T.B.D.); Dave.Kawadza@nwu.ac.za (D.T.K.); katlego.sehlabane@gmail.com (K.S.S.); mutsamt@yahoo.com (M.M.T.); 2 College of Natural, Applied and Health Sciences, Wenzhou-Kean University, 88 Daxue Road, Wenzhou 325060, Zhejiang Province, China; alwong@kean.edu; 3 Department of Chemistry, Biology & Biotechnology, University of Perugia, Borgo XX Giugno 74, 06121 Perugia, Italy; christophandreas.gehring@unipg.it

**Keywords:** *Arabidopsis thaliana*, adenylate cyclase, cAMP, clathrin assembly, endocytosis, pathogen responses

## Abstract

Adenylate cyclases (ACs), much like guanylate cyclases (GCs), are increasingly recognized as essential parts of many plant processes including biotic and abiotic stress responses. In order to identify novel ACs, we have applied a search motif derived from experimentally tested GCs and identified a number of *Arabidopsis thaliana* candidates including a clathrin assembly protein (AT1G68110; AtClAP). AtClAP contains a catalytic centre that can complement the AC-deficient mutant *cya*A in *E. coli*, and a recombinant AtClAP fragment (AtClAP^261–379^) can produce cyclic adenosine 3′,5′ monophosphate (cAMP) from adenosine triphosphate (ATP) in vitro. Furthermore, an integrated analysis of gene expression and expression correlation implicate cAMP in pathogen defense and in actin cytoskeletal remodeling during endocytic internalization.

## 1. Introduction

There has long been an extensive body of evidence, direct and indirect, that suggests that cyclic mononucleotide phosphates (cMNPs), including cyclic adenosine 3′,5′ monophosphate (cAMP), have important roles in many plant processes (for review see [1,2,3]). We are also beginning to understand cMNP functions at the single-molecule level, examples being the modulation of calcium channels in the plasma membrane of *Arabidopsis* leaf guard and mesophyll cells by cAMP [4] or the effect of guanosine 3′,5′ monophosphate (cGMP) on the kinase activity of receptor kinases [5,6]. At the systems level, a number of plant downstream cMNP targets have recently been identified experimentally [7] and they include cMNP-binding proteins that function as key enzymes in the Calvin cycle and photorespiration pathway.

The presence of cAMP and its role as a signalling molecule in plant cells is not in question; however, knowledge of the purine nucleotide cyclases responsible for its synthesis still remains elusive and poorly described. To date, only five plant adenylate cyclases (ACs) have been identified—the *Zea mays* pollen-signaling protein (ZmPSiP; AJ307886), responsible for pollen tube growth and reorientation [8]; the *Arabidopsis thaliana* pentatricopeptide repeat protein (AtPPR-AC; AT1G62590) [9]; the *Nicotiana benthamiana* adenylyl cyclase protein (NbAC; ACR77530), responsible for tabtoxinine-β-lactam-induced cell death and the occurrence of wildfire disease [10]; the *Hippeastrum hybridum* adenylyl cyclase protein (HpAC1; ADM83595), involved in stress signaling [11] and the *Arabidopsis thaliana* K^+^-uptake permease (AtKUP7; AT5G09400) [12].

The identification of ACs in *Arabidopsis* involved undertaking an in silico systematic approach that consists of both the prediction and experimental testing of candidate molecules. Prediction involved the identification of key amino acid residues in the catalytic centre of known and experimentally tested guanylate cyclases (GCs) and ACs [1,13]. These motif searches, supported by structure modelling [1,14], have proven successful in the identification of AtPPR-AC [9] and AtKUP7 [12]. Incidentally, the same consensus motif sequences found in AtPPR-AC and AtKUP7 are also present in the ZmPSiP and NbAC—two AC molecules with confirmed biological functions in plants [8,10]. Therefore, in order to advance our further understanding of plant ACs and their cAMP-dependent signalling, we have used the same systematic approach to predict and experimentally test an additional AC candidate from *Arabidopsis thaliana*. The AC search motif was based on the GC motif used in the identification of several functional GC centres by replacing the amino acid at position 3 of the motif to [DE] in order to give preference to adenosine triphosphate (ATP) rather than the guanosine-5′-triphosphate (GTP) substrate. This substitution of the residue that confers substrate specificity has been previously shown to successfully identify plant ACs [15,16]. We also note that since the discovery of the first GC, AtGC1, in 2003 [13], several GC ([3], for review see) and AC centres have been identified in AtPPR-AC [9], AtKUP7 [12], ZmPSiP [8] and NbAC [10]. Importantly, these functional catalytic centres have been shown to play important signalling roles e.g., acting as intramolecular cross-talks between catalytic activities or as molecular switches [5,17] and have varying biological implications [6,18,19]. It is now widely agreed that there are two groups of GCs, one the canonical GC domains in often stand-alone molecules and the other, GC centres found usually in multi-domain protein complexes [20]. Here we show that an *Arabidopsis* clathrin assembly (AtClAP; AT1G68110) protein harbours an active AC activity and we interpret this finding in the context of the role of cAMP in clathrin function and endocytosis.

## 2. Results

### 2.1. Identification of an Adenylate Cyclase Catalytic Centre at the Cytosolic Region of the AtClAP

**Figure 1:**
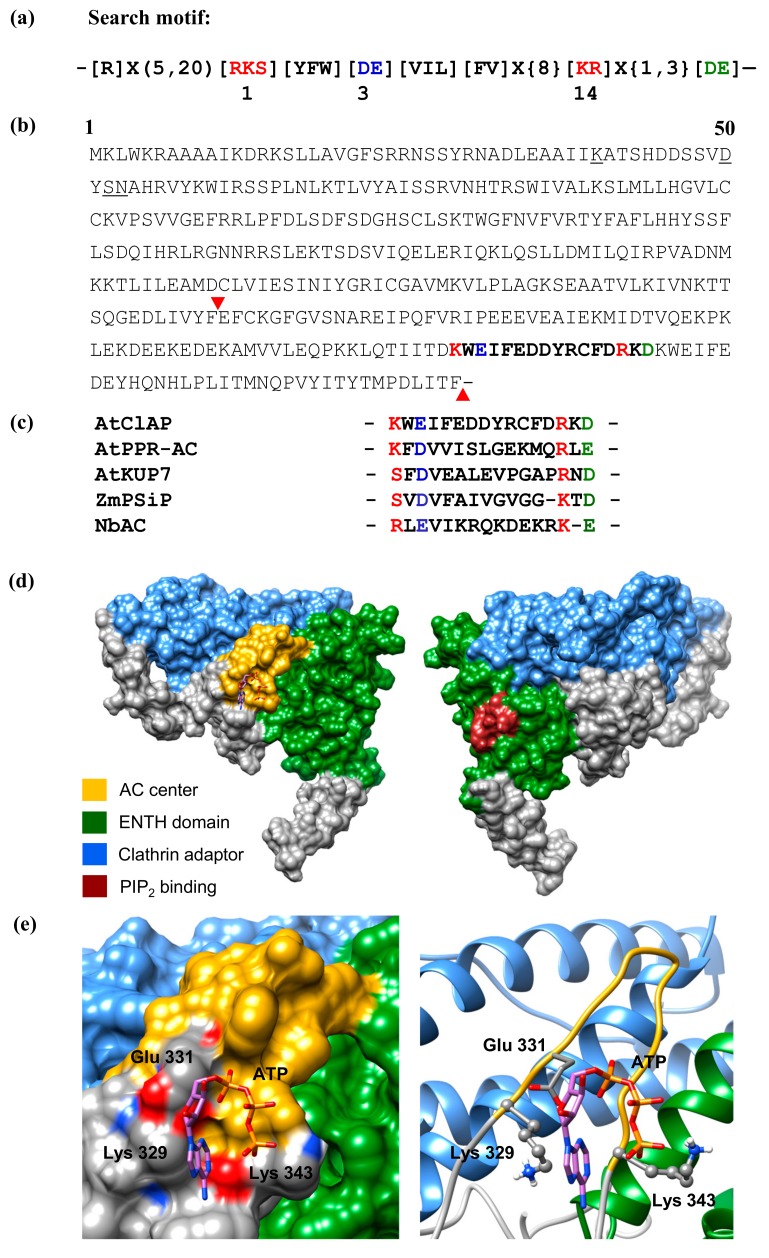
(**a**) The 14 amino acid adenylate cyclase (AC) search motif derived from annotated and experimentally tested guanylate cyclase (GC) and AC catalytic centres. The residue forming hydrogen bonding with purine at position 1 is highlighted in red, the residue conferring substrate specificity in position 3 is highlighted in blue, while the aa in position 14, stabilizing the transition state from ATP to cAMP, is highlighted in red. The amino acid [DE] at 1–3 residue downstream from position 14, participates in Mg^2+^/Mn^2+^-binding and is coloured green. *Arabidopsis thaliana* candidates including AtClAP were retrieved with the motif devoid of the sequences upstream of position 1; (**b**) The complete amino acid sequence of AtCIAP with the AC catalytic centre highlighted in bold and the 119 amino acid fragments tested for AC activity indicated within the inverted red triangles. The underlined amino acids mark an N-terminal phosphatidylinositol 4,5-bisphosphate (also referred to as PtdIns(4,5)*P*_2_, PIP_2_ or PI(4,5)P_2_) binding site; (**c**) Alignment of the AC centres of NbAC (ACR77530), ZmPSiP (AJ307886), AtKUP7 (AT5G09400), AtPPR-AC (AT1G62590) and AtClAP (AT1G68110); (**d**) Full-length models of AtClAP with the AC centre (gold) and the PIP_2_ binding site (brown) represented in the left and right panels. The Epsin N-terminal homology (ENTH) and clathrin adaptor domains are shown as green and blue, respectively, and they both make up the membrane-binding region of the protein; (**e**) Docking of ATP at the AC centre and the interaction of ATP with the key residues at the catalytic centre of AtClAP shown as surface (left) and ribbon (right) models. The residues implicated in interactions with ATP are coloured Ca according to their charges and shown as individual atoms in the ribbon model. AtCIAP was modeled using the iterative threading assembly refinement (I-TASSER) method [22] and ATP docking simulation was performed using AutoDock Vina (ver. 1.1.2) [23].

In order to identify candidate plant ACs, we have modified the GC search motif [21] at position 3, changing it from [CTGH] to [DE] (Figure 1a).

This substitution is based on previous findings which indicated that the conversion of GCs into ACs and vice versa could be achieved by a single mutation in the aa that confers substrate specificity [15,16]. When the *Arabidopsis* proteome is queried with the AC motif ([RKS][YFW][DE][VIL]X{9}[KR]X{1,3}[DE]), 159 proteins are retrieved and 77 when an [R] between the 5th and 20th aa upstream of position 1 is included. Since it is likely that this core AC motif may have identified false positives in what seems to be a large number of hits (159), we have included an [R] between the 5th and 20th aa upstream of position 1 as an additional filter to obtain a narrower list of hits. [R] in this position is known to be involved in pyrophosphate binding in class III but not class II GCs [16] and 77 hits were obtained when this is included as an additional filter. However, we decided to omit the [R], since it might be substituted by a lysine [K], which also contains a charged side chain. After omitting [R], we instead included [FV] at position 5 of the motif, which is a common feature in experimentally tested plant GCs (e.g., [18,24,25]) (Figure 1a), to obtain a narrower and more inclusive list of hits with greater confidence. This motif retrieved 10 proteins including AT1G68110 (Figure 1b). We have also noted that the core motif with just the functionally assigned residues ([RKS]X[DE]X{10}[KR]X{1,3}[DE]) is present in many annotated plant molecules (Figure 1c), including clathrins.

In addition to the identification of an AC catalytic centre in AtCIAP using a rationally designed AC search motif, we also used computational methods to assess the feasibility of this AC centre to bind the substrate ATP and catalyse the subsequent conversion into cAMP. We have modelled the full-length AtClAP by iterative threading and showed in this model that the AC catalytic centre is solvent-exposed, thus allowing for unimpeded substrate interactions and presumably catalysis (Figure 1d). Further probing of the AC centre by molecular docking of ATP suggests that the amino acid residue that stabilizes the transition state from ATP to cAMP in AtClAP is conferred by the residue at position 15 rather than the residue at position 14 as observed in AtKUP7 (Figure 1e) [12] and other experimentally confirmed plant GC centres [14]. If we add an additional [KR] at position 15 ([R]X{5,20}[RKS][YFW][DE][VIL]X{8}[KR][KR]X{0,2}[DE]) to the AC search motif to allow for the identification of similar candidate AC centres, we retrieve 11 proteins from the *Arabidopsis* proteome.

### 2.2. AtClAP Rescues an Adenylate Cyclase Deficient E. coli Mutant Strain

In order to investigate if the AtCIAP AC catalytic centre can rescue an *E. coli* AC-deficient mutant, fragment AtCIAP^261–379^ was cloned and expressed in an *E. coli* SP850 strain lacking the AC (*cya*A) essential for lactose fermentation.

**Figure 2:**
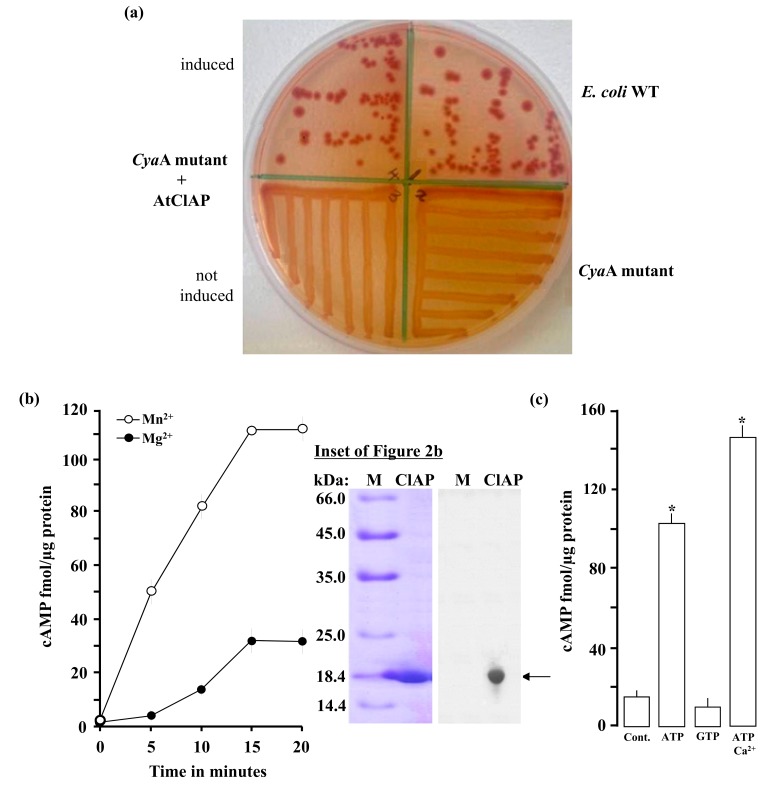
(**a**) The recombinant AC domain of AtCIAP^261–379^ complemented the *cya*A mutant *E. coli* (SP850). The wild-type *E. coli* shows a strong reddish colour while both the *cya*A mutant and the *cya*A mutant with an un-induced recombinant AtCIAP^261−379^ yielded yellowish colonies; (**b**) Cyclic AMP generated by the recombinant AtCIAP^261–379^ at different time points in reaction mixes containing at final concentrations, 5 µg protein, 1 mM ATP and 5 mM Mn^2+^ or Mg^2+^. Inset: A Coomassie brilliant blue-stained gel after resolution of the affinity purified His-tagged recombinant AtCIAP^261−379^ (arrow) by sodium dodecyl sulfate–polyacrylamide gel electrophoresis (SDS-PAGE) (left plane) and a Western blot analysis of the resolved AtCIAP^261−379^ with an anti-HIS antibody (right plane); (**c**) Cyclic AMP generated by 5 µg AtCIAP^261−379^ in the presence (at final concentrations) of 1 mM ATP or GTP, or 1 mM ATP and 250 µM Ca^2+^ when 5 mM Mn^2+^ ion is the cofactor (control reaction contained all other components except the protein and Ca^2+^). Data are mean values (n = 3) and error bars show standard error (SE) of the mean. Asterisks denote values significantly different from those of control (*p* < 0.05) determined by analysis of variance (ANOVA) and *post hoc* Student–Newman–Keuls multiple range tests.

As a result of the *cya*A mutation, the AC deficient and un-induced transformed *E. coli* cells remain yellowish in colour when grown on MacConkey agar. In contrast, the AtCIAP^261−379^ transformed *E. coli* SP850 cells, when induced with 0.5 mM IPTG, form deep reddish colonies much like the wild-type *E. coli* (Figure 2a) thus indicating a functional AC centre in the recombinant AtCIAP^261−379^.

### 2.3. In Vitro Adenylate Cyclase Activity of Recombinant AtClAP

**Figure 3:**
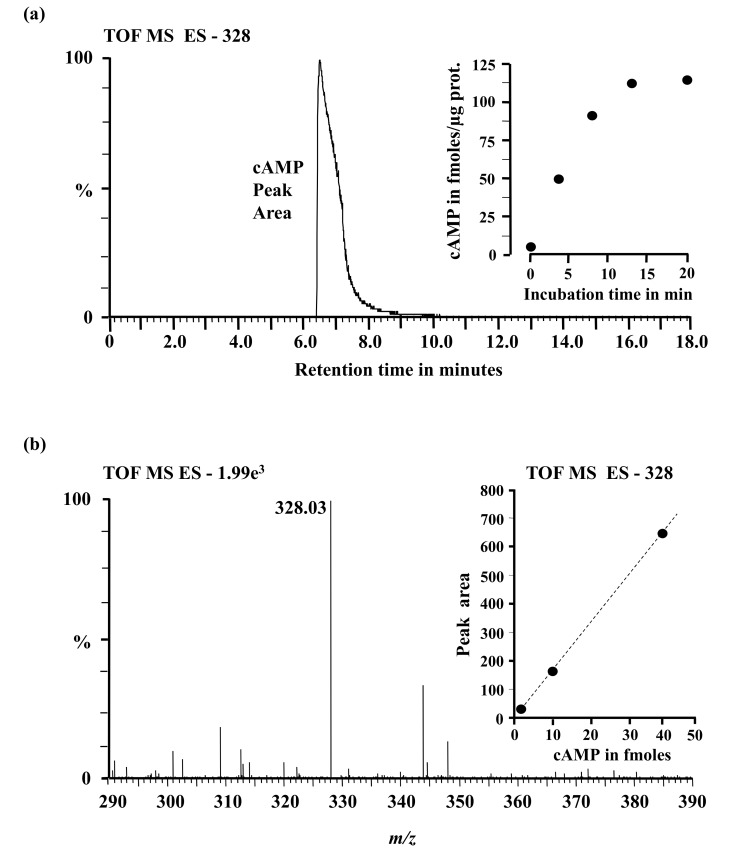
(**a**) An extracted mass chromatogram of the *m*/*z* 328 [M-1]^−1^ ion of cAMP generated by 5 µg of the AtCIAP^261−379^ in a reaction system containing at final concentrations, 50 mM Tris-Cl; pH 8.0, 1 mM ATP and 5 mM Mn^2+^ after 20 min. Inset: Incubation time course; (**b**) Mass of the resultant peak in the chromatogram. Inset: Detection calibration curve.

To test if the AtCIAP AC catalytic centre can generate cAMP in vitro, the AtCIAP^261−379^ was expressed in *E. coli* and affinity purified (Figure 2b, inset). The AC activity of the purified recombinant was then tested in a reaction mixture containing ATP and/or GTP as substrate, Mn^2+^ or Mg^2+^ as the cofactor, and Ca^2+^ as a modulator, followed by measurement of cAMP by enzyme immunoassay. Maximum activity was reached after 15 min of the reaction system, generating about 110 fmols/µg protein of cAMP in the presence of Mn^2+^ and approximately 31 fmols/µg protein of cAMP in the presence of Mg^2+^ (Figure 2b). The recombinant AtCIAP^261−379^ has a substrate preference for ATP rather than GTP and is Mn^2+^-dependent with its activity significantly enhanced by Ca^2+^ (Figure 2c). Incidentally, Ca^2+^ dependent increases of plant GC have been observed previously [5] and shown to be the switch between kinase and GC activities in the Phytosulfokine receptor (AtPSKR1). The molecular mechanism through which this Ca^2+^ dependence occurs is not currently known, but it is conceivable that Ca^2+^ invokes structural changes to the AC center, thereby enhancing catalytic activity. Cyclic AMP was also measured by mass spectrometry, another method capable of specifically and sensitively detecting cAMP levels at femtomolar concentrations. This second method confirmed presence of cAMP in the reaction samples (Figure 3), thereby validating the enzyme immunoassay technique and confirming function for the AtCIAP AC catalytic centre.

### 2.4. Inferring Function from AtClAP Transcriptional Data

When analysed for co-expression of the *AtClAP* gene (At1g68110), we noted that the 200 most correlated genes (ECG200) have high r values of >0.7 (Table S1). These expression-correlated genes are also significantly enriched for the “biological process” gene ontology (GO) categories “vesicle mediated transport” and “vesical fusion” and for the “molecular function” GO category, enrichment occurs in the categories “soluble N-ethylmaleimide-sensitive fusion attachment protein (SNAP) receptor activity” and “soluble N-ethylmaleimide-sensitive factor attachment protein receptor (SNARE) binding” (PANTHER Version 12.0 (released 10 July 2017)) [26]. SNAP receptor activity refers to a specific membrane interaction with one or more SNAREs on another membrane to mediate membrane fusion. This is entirely consistent with the annotation of *AtClAP* as an ENTH (Epsin NH_2_ terminal homology)/ANTH/VHS superfamily protein with a role in clathrin assembly and endocytosis.

**Figure 4:**
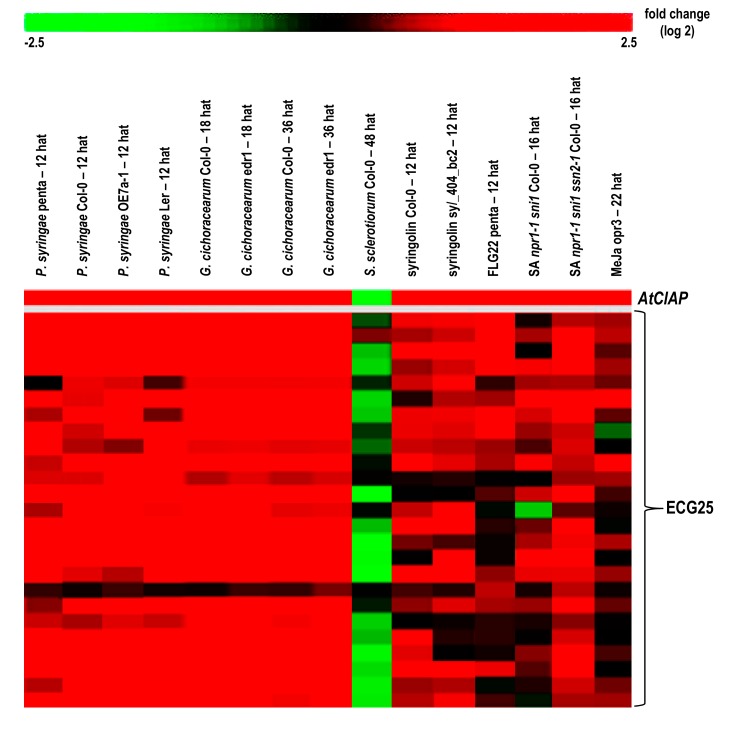
The heatmap constructed to illustrate the fold change (log2) in expression of *AtClAP* and 25 selected expression-correlated genes (ECG25) in response to selected microarray experiments. The experiments presented include; *P. syringae* (12 h after treatment (hat), GSE17464 and E-MEXP-1094), *G. cichoracearum* (18 and 36 hat, GSE26679), *S. sclerotiorum* (48 hat, E-MEXP-3122), syringolin (12 hat, AT-00258/FGCZ and E-MEXP-739), flg22 (12 hat, GSE17464), salicylic acid *npr1-1 sni1* double and *npr1-1 sni1 ssn2-1* triple mutants (16 hat, GSE23617) and methyl jasmonate (22 hat, GSE17464). Details of the microarray experimental conditions are presented in Appendix D.

When we extended the analysis to identify conditions that induce *AtClAP* and the expression of correlated genes (Table S1), we noted induction by biotrophic pathogens [27] e.g., *Pseudomonas syringae* and the associated pathogen effector molecules (syringolin A and flagellin 22) (Figure 4).

These genes are also up-regulated in the *npr1-1 sni1* double and *npr1-1 sni1 ssn2-1* triple mutants. The *npr1-1* is a mutant with defects in the *NPR1* gene [28] that controls the onset of systemic acquired resistance (SAR) that is dependent on salicylic acid signaling (SA) [29]. The *sni1* and *ssn2-1* are both regulators of the SAR that suppress effects of the *npr1-1* by triggering expression of the *PR* genes and conferring resistance to pathogens [30,31].

## 3. Discussion

Our study on AtClAP deals with AC functional centres identified initially from a motif-based approach using the catalytic centres of canonical GC domains as a guide. It is likely that this new class of GC/AC centres [32] have different activations/regulations since they do not contain the full GC domain but are often embedded within proteins with other primary functions, hence structurally dissimilar. In canonical GCs/ACs, the catalytic site may comprise of regions from two separate proteins, which dimerise to form the active site [33]. Since plant proteins exist as multi-domain complexes, the overall structure is entirely different from canonical GCs/ACs as they assume folds that reflect their primary functions e.g., as receptors or protein kinases. However, they can accommodate functional GC/AC catalytic centres at moonlighting sites usually embedded within larger domains. Our motif, whether ACs or GCs, was built to include conserved residues at catalytic sites of canonical GCs/ACs regardless of which chain of the dimers they may have come from. These functional centres have since been shown to exist in complex molecules with varying biological functions, ranging from ion transport in AtKUP7 to peptide and hormone perceptions in AtPepR1 and AtPSKR1 or AtBRI1 (for review see [2]). This suggests that the nature of such functional centres is to provide a tailored localized cellular-signalling role but with broad biological significance. In this study, AtClAP which is a clathrin assembly protein with annotated Epsin N-terminal homology (ENTH), PIP_2_ binding and clathrin adaptor domains, may exhibit a similar localized signaling role through its AC centre.

As a protein that is involved in endocytosis, AtClAP requires ATP existing in complex with heat-shock proteins (e.g., Hsc 70) for the release of clathrin coat and adaptors that are bound to the vesicles [34]. Correspondingly, the ATPase subunit AT1G20880 is represented in the *AtClAP*-ECG200. In addition, genes encoding for components related to endocytosis such as vesicle transport and membrane proteins (e.g., AT1G26670, AT4G32150, AT4G15780, AT3G22290, AT5G47180 and AT5G22360) are also represented in the ECG200, thus supporting the primary role of AtClAP in clathrin-dependent endocytosis. More interestingly, our computational analysis identified a functional AC centre in AtClAP that is located on a solvent-exposed region at the C-terminal of the protein. This region is separate from the membrane-binding ENTH and clathrin adaptor regions and faces towards the cytoplasm, thus indicating functions unrelated to membrane binding. Our structural analysis, including the evaluations of 3D models and ligand-docking simulations, suggest that this AC centre is able to accommodate ATP in configurations reminiscent of another recently identified AC centre in AtKUP7 [12]. Here, we have shown that the recombinant fragment AtClAP^261–379^ not only generated cAMP in vitro but is also capable of generating cAMP endogenously (Figure S1; Appendix C) and rescuing an AC-deficient *E. coli* (Figure S2), thus demonstrating the functionality of this AC centre. The generation of cAMP at this auxiliary site of AtClAP therefore prompted speculation for a role of cAMP in clathrin-mediated endocytosis. Indeed, cAMP has been shown to be essential for actin polymerization and the general regulation of actin dynamics in the plant cell [35] especially in fast-growing tissues such as the pollen tube [36]. Correspondingly, Actin-8 (AT1G49240) has expression that is co-related with *AtClAP* (Table S1). Our findings have, therefore, identified a novel component that is seemingly missing in the clathrin-dependent endocytosis-signaling cascade of plant cells. Generation of cAMP by the AC centre in AtClAP is a secondary role essential for the actin-driven regulation of clathrin-coated pit dynamics at the plasma membrane.

**Figure 5:**
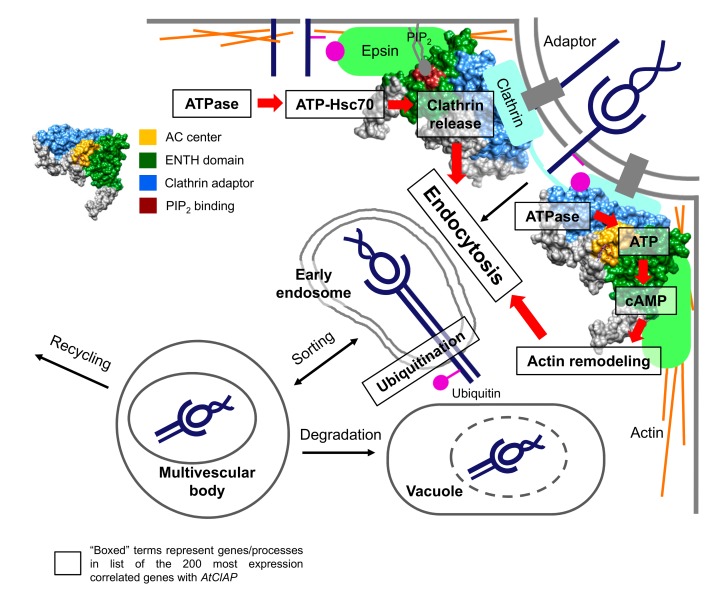
A model illustrating the dual-role of AtClAP in clathrin-mediated endocytosis. In the primary function, AtClAP assembles clathrin at protein adaptors attached to the membrane of a newly forming vesicle and recruits components essential for endocytosis such as Epsin and other accessory proteins to internalize membrane proteins, effectors or receptor–ligand complexes. ATPases generate ATP, which combines with heat shock proteins (e.g., Hsc70) to release the bound clathrin and adaptors during invagination. In the secondary role, ATP is converted to cAMP by the solvent-exposed AC centre of AtClAP and cAMP, in turn, assembles actin to aid endocytosis and to assist vesicle translocation in the cell. Internalized cargoes are transported into early endosomes, where they may be recycled to the membrane or tagged by ubiquitin for endosomal sorting and degradation in vacuoles. The ubiquitin tagged effectors may also be targeted for degradation through the ubiquitin-proteasome system. Boxed terms represent processes or genes that are found in the list of top-200 most expression correlated genes with *AtClAP*.

cAMP generated from the AtClAP AC centre not only organizes actin cytoskeleton to aid invagination of the cargo-containing vesicle but also for the translocation of the internalized vesicle into early endosomes by cytoskeletal reorganization [37]. In addition to Actin-8, myosin and myosin-binding proteins such as AT1G28410 and AT5G06560 are represented in the *AtClAP*-ECG200. The role of AtClAP in endocytosis is further consolidated when ubiquitin-related genes such as E3 ligases, RING-type ubiquitin E3 transferases and ubiquitin-conjugating enzyme E2 (e.g., AT5G32440, AT5G64920, AT4G27880, AT4G12570, AT5G40190, AT1G64230, AT5G41560, AT4G39910 and AT4G24990) are represented in the ECG200, therefore placing AtClAP in a protesome-dependent degradation pathway of effectors such as those observed during pathogen interactions [38]. This is consistent with our condition-specific analysis of AtClAP that identified induction by biotrophic pathogens (e.g., *P. syringae*) and effector molecules (syringolin A and flagellin 22). The cargo of internalized vesicles can be recycled to membrane or tagged by ubiquitin for endosomal sorting and degradation in vacuoles [37]. In the latter, vacuole-related proteins (AT1G26670, AT2G45980, AT1G16240, AT5G06140 and AT4G22750) and proteins related to Golgi transport (AT1G15880 and AT4G38260), proteolysis (AT1G05840) and autophagy (AT5G17290) are represented in the *AtClAP*-ECG200, thus supporting a vacuolar degradation role. A model depicting the dual-role of AtClAP in clathrin-mediated endocytosis is illustrated in Figure 5.

In summary, the *Arabidopsis thaliana* clathrin assembly protein (AT1G68110; AtClAP) contains a functional AC catalytic centre. Here we make a case for a role of this domain in both endocytosis and responses to pathogens. Future experiments will be focusing on establishing the nature of the link between cAMP, endocytosis and responses to pathogens.

## 4. Materials and Methods 

### 4.1. Generation of Recombinant AtClAP^261–379^

Total RNA was extracted from six-week old *Arabidopsis thaliana* ecotype Columbia-0 (Col-0) seedlings using the RNeasy plant mini kit, in combination with DNase 1 treatment, as instructed by the manufacturer (Qiagen, Crawley, UK). The copy DNA (cDNA) sequence of AtClAP was retrieved from The Arabidopsis Information Resource (TAIR) (https://www.arabidopsis.org, Phoenix Bioinformatics, Fremont, CA, USA) [39] and verified for presence of the AC catalytic centre using the PROSITE database located within the Expert Protein Analysis System (ExPASy) proteomics server (https://www.expasy.ch/) [40]. AtCIAP^261−379^ cDNA synthesis from the total RNA and subsequent amplification of the AtCIAP gene fragment from the cDNA were simultaneously performed in the presence of two sequence-specific primers (forward: 5′-GAATTCTGCAAAGGTTTCGGTGTCTCGAAC-3′ and reverse: 5′-GAATGTAATCAAATCTGGCATTGTATAAGT-3′ using a Verso 1-Step reverse transcription–polymerase chain reaction (RT–PCR) kit and in accordance with the manufacturer’s instructions (Thermo Scientific, Rockford, IL, USA). The PCR product was then cloned into a pTrcHis2-TOPO expression vector via the TA cloning system (Invitrogen Corp., Carlsbad, CA, USA) to make a pTrcHis2-TOPO:AtCIAP-AC fusion expression construct with a C-terminus His purification tag. Expression, purification and refolding processes of the recombinant AtCIAP^261−379^ protein were undertaken as is detailed elsewhere [17,18] and in Appendix A. The relative molecular mass of the AtCIAP^261−379^ was estimated using the ProtParam tool on the ExPasy Proteomics Server (http://au.expasy.org/tool/.protpatram.html) [40]. The purified protein was then used for in vitro enzymatic assays.

### 4.2. Gel Electrophoresis and Western Blotting Analysis of AtClAP

The purified AtClAP-AC recombinant was resolved by sodium dodecyl sulfate–polyacrylamide gel electrophoresis (SDS-PAGE) on a 12% (*v*/*v*) gel followed by staining with Coomassie brilliant blue (Sigma-Aldrich Corp., St. Louis, MI, USA). For Western analysis, the resolved protein was transferred to a polyvinylidene fluoride (PVDF) membrane (Hybond-P; GE Healthcare, Buckinghamshire, UK) by the semi-dry system (Bio-Rad Laboratories Inc., Hercules, CA, USA) in 25 mM Tris-Cl, 192 mM glycine buffer (pH 8.3) with 20% (*v*/*v*) methanol. After blocking in tris-buffered saline (TBS, 20 mM Tris, 100 mM NaCl) containing 3% (*w*/*v*) non-fat dry milk, the membrane was incubated with goat polyclonal anti-HIS antibodies (1:15,000; Sigma-Aldrich Corp., St. Louis, MI, USA). The blot was visualized with a horseradish peroxidase-conjugated secondary anti-goat IgG (1:60,000; Sigma-Aldrich Corp., St. Louis, MI, USA) for the histidine (HIS) tag, and detected by chemiluminescence.

### 4.3. Computational Assessment of the AtClAP Centre

An AtClAP model was generated using the iterative threading assembly refinement (I-TASSER) method [22]. The full-length AtClAP amino acid sequence was submitted to the I-TASSER server (http://zhanglab.ccmb.med.umich.edu/I-TASSER/) and the model with the highest quality based on its C-score, was downloaded from the server. The AtClAP model was visualised and analysed, and its images created using UCSF Chimera (v.1.10.1.) [41]. Docking of ATP to the AC centre of the AtClAP model was performed using AutoDock Vina (v.1.1.2) [23]. The ATP docking pose was analysed and the associated images created with PyMOL (v.1.7.4.) (The PyMOL Molecular Graphics System, Schrödinger, LLC).

### 4.4. In Vitro Adenylate Cyclase Enzymatic Assay and Detection of cAMP

The AC activity of the purified recombinant AtCIAP^261−379^ was determined in vitro by incubating 5 µg of the protein in 50 mM Tris-Cl buffer (pH 8.0), containing at final concentration, 5 mM Mg^2+^ or 5 mM Mn^2+^, 1 mM ATP and/or 1 mM GTP, followed by measurement of the generated cAMP. Since Ca^2+^ has been shown to enhance GC activity and, indeed, act as a cellular switch between the GC and kinase activities of AtPSKR1 [5], it was also tested against the AtCIAP^261−379^ at a final concentration of 250 µM and in the presence of 5 mM Mn^2+^ and 1 mM ATP. Levels of the generated cAMP were determined by enzyme immunoassaying following its acetylation protocol as described by the supplier’s manual (Sigma-Aldrich Corp., St. Louis, MI, USA; code: CA201). The methods are detailed elsewhere [12] and in Appendix B.

### 4.5. Mass Spectrometry Analysis of cAMP

The generated and acetylated cAMP samples were introduced into a Waters API Q-TOF Ultima mass spectrometer (Waters Microsep, Johannesburg, RSA) with a Waters Acquity UPLC at a flow rate of 180 mL/min. Separation was then achieved in a Phenomenex Synergi (Torrance, CA) 4 µm Fusion-RP (250 × 2.0 mm) column when a gradient of solvent “A” (0.1% (*v*/*v*) formic acid) and solvent “B” (100% (*v*/*v*) acetonitrile) was applied over 18 min. During the first 7 min, the solvent composition was kept at 100% (*v*/*v*) “A” followed by a linear gradient of up to 80% (*v*/*v*) “B” for 3 min, and then a re-equilibration to the initial conditions. An electrospray ionization in the negative (W) mode was used at a cone voltage of 35 V, to detect molecules and generate chromatograms.

### 4.6. Complementation of cyaA Mutation in the E. coli Adenylate Cyclase-Deficient Strain

The *E. coli cya*A mutant SP850 strain (lam-, el4-, relA1, spoT1, *cya*A1400 (:kan), thi-1) [42], deficient in the adenylate cyclase (*cya*A) gene, was obtained from the *E. coli* Genetic Stock Centre (Yale University, New Haven, CT, USA) (accession No. 7200). The strain was prepared to be chemically competent followed by its transformation with the pTrcHis2-TOPO:AtCIAP-AC fusion construct (through heat shock at 42 °C for 2 min). The transformed bacteria were then grown at 37 °C in LB media containing ampicillin (100 µg/mL) and kanamycin (15 µg/mL) until their cell density had reached an OD_600_ of 0.5. The cells were treated with 0.5 mM isopropyl-β-d-thiogalactopyranoside (IPTG, Sigma-Aldrich Corp., St. Louis, MI, USA) for transgene induction and further incubated for 4 h prior to streaking on crystal violet containing MacConkey agar.

### 4.7. Statistical Analysis

All data of immunoassay in this work was subjected in triplicates (n = 3) to analysis of variance (ANOVA) (Super-Anova, Statsgraphics Version 7, Statsgraphics Corp., The Plains, VI, USA). Where ANOVA revealed significant differences between treatments, means were separated by the *post hoc* Student–Newman–Keuls (SNK) multiple range test (*p* < 0.05).

## Supplementary Materials

The following are available online at http://www.mdpi.com/2218-273X/8/2/15/s1.

## Appendix A. Expression, Purification and Refolding of the Recombinant AtCIAP-AC261–379 Protein

For expression of the recombinant AtClAP^261–379^ protein, competent *E. coli* EXPRESS BL21 (DE3) pLysS DUOs cells (Lucigen Corp., Middleton, WI, USA) were transformed with the pCRT7/NT-TOPO:AtClAP-AC fusion construct and grown in double-strength yeast-tryptone media (16 g/L tryptone, 10 g/L yeast extract, 5 g/L NaCl and 4 g/L glucose; pH 7.0) containing 100 µg/mL ampicillin and 34 µg/mL chloramphenicol, in an orbital shaker (250 rpm) at 37 °C. Protein expression was induced by the addition of isopropyl-β-d-thiogalactopyranoside (IPTG, Sigma-Aldrich Corp., St. Louis, MI, USA) to a final concentration of 1 Mm and when the optical density (OD_600_) of the cell culture had reached 0.5 (approximately 3 h). The culture was then left to grow for a further 3 h at 37 °C.

The recombinant AtClAP^261–379^ was purified by preparing a cleared cell lysate of the induced *E. coli* cells under non-native denaturing conditions, where the harvested cells were re-suspended in lysis buffer (8 M urea, 100 Mm NaH_2_PO_4_, 10 Mm Tris-Cl; Ph 8.0, 500 Mm NaCl, 20 Mm β-mercaptoethanol, 7.5% (*v*/*v*) glycerol) at a ratio of 1 g pellet weight to every 10 Ml buffer volume, mixed thoroughly using a mechanical stirrer at 24 °C for 1 h and then centrifuged at 2500× *g* for 15 min. The supernatant was collected as the cleared lysate and transferred to 2 Ml of 50% (*w*/*v*) Ni-NTA slurry (Sigma-Aldrich Corp., St. Louis, MI, USA) that had been pre-equilibrated with 10 Ml of lysis buffer and then gently mixed on a rotary mixer for 1 h at 24 °C. The lysate-resin mixture was loaded into an empty XK16 column (Bio-Rad Laboratories Inc., Hercules, CA, USA), allowed to settle and the flow-through discarded. The resin was washed three times with 30 Ml wash buffer (8 M urea, 100 Mm NaH_2_PO_4_, 10 Mm Tris-Cl; Ph 8.0, 500 Mm NaCl, 20 Mm β-mercaptoethanol, 7.5% (*v*/*v*) glycerol, and 40 Mm imidazole).

Subsequently, the resins were equilibrated with 2 Ml gradient buffer (8 M urea, 200 Mm NaCl, 50 Mm Tris-Cl; Ph 8.0 and 20 Mm β-mercaptoethanol) before the column was connected to a BioLogic DuoFlow chromatography system (Bio-Rad Laboratories Inc., Hercules, CA, USA) programmed to run a linear refolding gradient. The refolding gradient for the denatured recombinant AtClAP^261–379^ was then performed by linearly diluting the 8 M gradient buffer to a 0 M urea concentration with a refolding buffer (200 Mm NaCl, 50 Mm Tris-Cl; Ph 8.0, 500 Mm glucose, 0.05% (*w*/*v*) poly-ethyl glycol, 4 Mm reduced glutathione, 0.04 Mm oxidized glutathione, 100 Mm non-detergent sulfobateine, and 0.5 Mm phenylmethanesulfonylfluoride (PMSF)) over 10 h at a flow rate of 0.5 Ml/min. After renaturation, the recombinant protein was eluted in 2 Ml of elution buffer (200 Mm NaCl, 50 Mm Tris-Cl; Ph 8.0, 250 Mm imidazole, 20% (*v*/*v*) glycerol, and 0.5 Mm PMSF). The eluted protein fraction was then de-salted and concentrated using a Spin-XUF filtration/concentration device and in accordance with the manufacturer’s instructions (Corning Corp., Corning, NY, USA) with a molecular weight cut-off point of 3000 Da. Protein concentration was determined by the Bradford method and a 2000 nanodrop spectrophotometer (Thermo Scientific Inc., Waltham, MA, USA) before the recombinant protein was stored at −20°C.

## Appendix B. In Vitro Cyclic Nucleotide Assays

The AC activity of the purified recombinant AtClAP^261–379^ protein was measured in vitro by incubating 5 µg of protein in 50 Mm Tris-Cl buffer (Ph 8.0), containing (at final concentration) 5 Mm Mg^2+^ or 5 Mm Mn^2+^, 1 Mm ATP and/or 1 Mm GTP, and or not supplemented with 250 µM CaCl_2_, in a final volume of 200 µL. Background Camp levels were measured in tubes that contained the incubation mediums but no protein and CaCl_2_. All incubations were performed at room temperature (24 °C) for 20 min and terminated by the addition of 10 Mm EDTA followed by boiling for 3 min and cooling on ice for 2 min before centrifugation at 2500× *g* for 3 min. The resulting supernatant was assayed for Camp content using the Camp-linked enzyme immunoassaying kit following its acetylation protocol and as is described by the supplier’s manual (Sigma-Aldrich Corp., St. Louis, MI, USA; code: CA201). The anti-cAMP antibody in this assaying system is highly specific for cAMP and has approximately a 10^6^ times lower affinity for cGMP. In all cases, each experiment was performed in triplicate (n = 3) using three different protein extracts that were independently expressed and purified.

## Appendix C. Endogenous Activity Assaying of the Recombinant AtCIAP-AC261−379 Protein

EXPRESS BL21 (DE3) pLysS DUOs cells (Lucigen Corp., Middleton, WI, USA) harbouring the AtClAP-AC gene fragment were grown in double strength yeast-tryptone media supplemented with 100 µg/mL ampicillin and 34 µg/mL chloramphenicol, in an orbital shaker (250 rpm) at 37 °C. At optical density (OD_600_) of 0.5 of the cells, the culture was split into two. In the first cell culture, 1 mM IPTG (to induce protein expression) together with 1 mM 3-isobutyl-1-methylxanthine (IBMX, to inhibit phosphodiesterases) were added while in the second culture (control), only 1 mM IBMX was added. The two cultures were then left to grow for a further 3 h at 37 °C at 250 rpm. At the end of the incubation period, cAMP was extracted from the cell cultures following an established extraction procedure for suspension cells (Amersham Healthcare, Buckinghamshire, UK; code: RPN226) and the resultant cAMP then measured by enzyme immunoassay following the acetylation protocol described in the supplier’s manual (Sigma-Aldrich Corp., code: CA201). In all cases, each experiment was performed in triplicate (n = 3) using three different samples that were independently extracted.

## Appendix D. Description of Microarray Experiments

*P. syringae pv tomato study 11, (penta)/untreated disc samples (penta) (GSE17464).* Leaf disc samples of five weeks old penta mutant plants infiltrated with *Pseudomonas syringae* pv tomato virulent DC3000 bacteria (2 × 10^5^ cfu/mL) were collected 12 h after treatment. Genotype: penta mutant (n = 3).

*Transcription profiling of leaves from wild type and AtFAAH (E-MEXP-1094).* Leaf samples from 4-week-old OE7a-1 (AtFAAH (AT5G64440) overexpression line) plants, inoculated with the non-host pathogen *Pseudomonas syringae* pv syringae for 12 h. Genotype: Transgenic Col-0 (n = 3).

*P. syringae pv tomato study 2, (OE7a-1)/untreated disc samples (OE7a-1) (E-MEXP-1094)*. Leaf samples from 4-week-old OE7a-1 (AtFAAH (AT5G64440) overexpression line) plants, inoculated with the non-host pathogen *Pseudomonas syringae* pv syringae for 12 h. Genotype: OE7a-1 (n = 3).

*P. syringae pv tomato study 11, (Ler)/untreated disc samples (Ler) (GSE17464)*. Leaf disc samples of five-week-old penta mutant plants infiltrated with *Pseudomonas syringae* pv tomato virulent DC3000 bacteria (2 × 10^5^ cfu/mL) were collected 12 h after the treatment. Genotype: Landsberg erecta (n = 3).

*G. cichoracearum study 3, (36 h) non-infected whole rosette samples (edr1) (GSE26679)*. Whole rosette samples of 4-week-old edr1 mutant plants collected 18 h and 36 h after inoculation with *Golovinomyces cichoracearum*. Genotype: edr1 (n = 2).

*G. cichoracearum study 2, (36 h) non-infected whole rosette samples (Col-0) (GSE26679).* Whole rosette samples of 4-week-old Col-0 plants collected 18 h and 36 h after inoculation with *Golovinomyces cichoracearum*. Genotype: Col-0 (n = 2).

*Transcription profiling after infection with Sclerotinia sclerotiorum (E-MEXP-3122)*. Col-0 plants were grown for 4–6 weeks (10h light (250–300 µmol photons m^−2^s^−1^)/14 h dark cycles; 22 °C, substrate:vermiculite to potting soil ratio (1:2)), then inoculated with the broad host range necrotrophic ascomycete *Sclerotinia sclerotiorum* isolate 1980 by placing an agar plug (1.6 mm in diameter) containing actively growing hyphal tips on the adaxial surface of a rosette leaf for 48 h. The *Sclerotinia sclerotium* treated leaves were then harvested for the microarray analysis. Genotype: Col-0 (n = 3).

*Syringolin study 3, solvent treated leaf samples (E-MEXP-739).* Primary leaf samples of uninfected Col-0 plants treated with syringolin A (syl A) solution (20 µM) for 12. Genotype: Col-0 (n = 3).

*Syringolin study 2, solvent treated leaf samples (AT-00258/FGCZ).* Arabidopsis mutant plants (syl_404_bc2) were sprayed with 20 µM syringolin A. Hybridization probes were prepared from primary leaves harvested 12 h after syringolin treatment. Genotype: syl_404_bc2 (n = 3).

*Arabidopsis mutants treated with flg22 (GSE17464).* Leaf disc samples of five-week-old penta mutant plants grown under short day (8 h light/16 h dark, 120 µmols^−1^m^−2^) conditions treated with 100 nM flg22 and collected after 12 h. Genotype: penta mutants (n = 3).

*The impact of SSN2 on plant defense genes (GSE23617).* Whole plant samples of 3-week-old *npr1-1 sni1* double mutant plants treated with 500 µM salicylic acid for 16 h. Genotype: Col-0 (n = 2).

*(Salicylic acid study 7, (npr1-1 sni1 ssn2-1)/solvent treated whole plant samples (GSE23617).* Whole plant samples of 3-week-old *npr1-1 sni1 ssn2-1* triple mutant plants treated with 500 µM salicylic acid for 16 h. Genotype: Col-0 (n = 2).

*Arabidopsis mutants treated with methyl jasmonate (GSE17464).* Stamens of *opr3* mutants were treated with 0.03% methyl jasmonate and stamens were harvested 22 h after treatment. Genotype: Col-0 (n = 3).
