# Supplementary material for: An Arabidopsis Clathrin Assembly Protein with a Predicted Role in Plant Defense Can Function as an Adenylate Cyclase
Source: Biomolecules. 2018 Mar 23;8(2):15. doi: 10.3390/biom8020015 (PMC6022867; doi:10.3390/biom8020015)
Supplement: Supplementary file 1 [file biomolecules-08-00015-s001.pdf]

Table S1. List of the top 200 expression correlated genes with *AtClAP* (AT1G68110).

Num	Locus and Gene Ontology terms[a]	r-value	Annotation and Functional Description
**1**	AT1G05840[PT, IPTI, PO, PF, EPLP, PLP, LM, FABO, PTP, MACP, IPTT, LO, PI, FAO, FACP, I-GV-MT, LT, VMT, LL, PTT, CLCP, PIPM, LIT, PP, VRTM]	0.81	Involved in proteolysis
**2**	AT1G58030[PT, IPTI, PO, PF, EPLP, PLP, LM, FABO, PTP, MACP, IPTT, LO, PI, FAO, FACP, CLCP, PIPM, PP, VRTM]	0.73	Catalase-2, Cationic amino acid transporter 2, vacuolar
**3**	AT2G28910[PT, IPTI, PF, EPLP, PLP, PTP, IPTT, PI, I-GV-MT,LT, VMT, LL, PTT, PIPM, LIT, PP, VRTM]	0.73	CAX-interacting protein 4
**4**	AT3G07560[PT, IPTI, PO, EPLP, PLP, LM, FABO, PTP, MACP, IPTT, LO, PI, FAO, FACP, I-GV-MT, LT, VMT, LL, PTT, CLCP. PIPM, LIT]	0.72	Peroxisomal membrane protein 13
**5**	*AT1G26670[PT, IPTI, EPLP, PLP, PLV, EPLV, PTP, PTV, IPTT, PI, I-GV-MT, LT, VMT, SB, SRA, LL, PTT, PIPM, LIT]	0.81	Vesicle transport V-SNARE family protein
**6**	*AT1G15880[PT, I-GVMT, PLP, EPLP, PTP, PO, IPTI, IPTT, PI, LT, VMT, SB, SRA, LL, PTT, PIPM, LIT]	0.80	Golgi snare 11
**7**	AT5G66160[PT, IPTI, PO, EPLP, PLP, PTP, IPTT, PI, I-GV-MT, LT, VMT, LL, PTT, PIPM, LIT]	0.73	Receptor homology region, transmembrane domain protein 1
**8**	AT1G64230[PT, IPTI, PO, EPLP, PLP, LM, FABO, PTP, MACP, IPTT, LO, PI, FAO, FACP, VMT, PTT, CLCP, PIPM]	0.74	Ubiquitin-conjugating enzyme E2 28
**9**	AT1G05790[PT, IPTI, PO, EPLP, PLP, LM, FABO, PTP, MACP, IPTT, LO, PI, FAO, FACP, ,PTT, CLCP, PIPM]	0.71	Lipase Class 3 family protein
**10**	AT3G19860[PT, IPTI, EPLP, PLP, LM, FABO, PTP, MACP, IPTT, LO, PI, FAO, FACP, PTT, CLCP, PIPM]	0.77	Transcription factor bHLH121
**11**	AT1G16240[PLV, EPLV, PTV, I-GV-MT, VMT, SB, SRA]	0.79	Syntaxin of plants 51
**12**	AT2G45980[PLV, EPLV, PTV, VMT]	0.80	ATG8-interacting protein 1
**13**	AT5G06140[PLV, EPLV, PTV, VMT]	0.78	Sorting nexin 1
**14**	*AT4G22750[PLV, EPLV, PTV, VMT]	0.77	Probable protein S-acyltransferase 13
**15**	AT5G66030[I-GV-MT, VMT]	0.74	Protein GRIP
**16**	*AT2G36900[VMT, SB, SRA]	0.81	Membrin 11
**17**	*AT4G32150[VMT, SB, SRA]	0.80	Vesicle-associated membrane protein 711
**18**	*AT4G17730[VMT, SB, SRA]	0.78	Syntaxin of plants 23
**19**	AT2G28370[VMT]	0.81	CASP-like protein 5A2

**20**	AT1G13450[VMT]	0.78	Trihelix tancription factor GT-1
**21**	AT1G49240[VMT]	0.77	Actin-8
**22**	AT4G24520[VMT]	0.75	NADPH-cytochrome P450 reductase 1
**23**	AT1G53400[VMT]	0.75	Uncharacterized protein
**24**	AT3G24315[VMT]	0.72	AtSec20 family protein
**25**	AT5G39590[VMT]	0.71	TLD-domain containing nucleolar protein
**26**	AT5G46410[VMT]	0.71	SCP1-like small phosphatase 4
**27**	AT1G26580[mRNACP, RNACP, PTT, N-TMCP]	0.77	Cpn60-beta4
**28**	AT5G12230[mRNACP, RNACP, N-TMCP]	0.76	Mediator of RNA polymerase II transcription subunit 19A
**29**	AT5G19330[mRNACP, RNACP, N-TMCP]	0.73	ARIA ARM REPEAT PROTEIN INTERACTING WITH ABF2
**30**	AT3G10030[mRNACP, RNACP, PF, N-TMCP, PP, VRTM]	0.75	Aspartate/glutamate/uridylate kinase family protein
**31**	AT2G45620[mRNACP, RNACP, PF, N-TMCP, PP, VRTM]	0.72	UTP:RNA uridylyltransferase 1
**32**	AT5G04910[PF, LM, FABO, MCP, LO,FAO, FACP, CLCP, PP, VRTM]	0.76	Peptidylprolyl isomerase
**33**	AT5G42820[PF, LL, PP, VRTM]	0.72	Splicing factor U2AF small subunit B
**34**	AT5G14080[PF, PP, VRTM]	0.79	Pentatricopeptide repeat-containing protein
**35**	*AT3G11100[PF, PP, VRTM]	0.78	Trihelix transcription factor ASIL1
**36**	AT5G20520[PF, PP, VRTM]	0.77	WAV2 alpha/beta-Hydrolases superfamily protein
**37**	AT4G38800[PF, PP, VRTM]	0.76	5'-methylthioadenosine/S-adenosylhomocysteine nucleosidase 1
**38**	AT3G14750[PF, PP, VRTM]	0.76	Protein FLX-like 1
**39**	AT2G26430[PF, PP, VRTM]	0.76	Cyclin-L11
**40**	AT3G01770[PF, PP, VRTM]	0.76	Transcription factor GTE11
**41**	AT1G12910[PF, PP, VRTM]	0.75	WD repeat-containing protein LWD1

**42**	AT3G47610[PF, PP, VRTM]	0.75	Transcription regulator/zinc ion binding protein
**43**	AT2G43430[PF, PP, VRTM]	0.73	Hydroxyacylglutathione hydrolase 1, mitochondrial
**44**	AT1G76460[PF, PP, VRTM]	0.72	RNA-binding (RRM/RBD/RNP motifs) family protein
**45**	AT1G15920[VRTM]	0.74	Probable CCR4-associated factor 1 homolog 2
**46**	AT2G33810[RB, VRTM]	0.72	Squamosa promoter-binding-like protein 3
**47**	AT3G17100[RB]	0.76	Transcription factor bHLH147
**48**	AT4G00720[RB]	0.74	Shaggy-related protein kinase theta
**49**	AT1G06390[RB]	0.74	Shaggy-related protein kinase iota
**50**	AT1G17120[RB]	0.74	Cationic amino acid transporter 8, vacuolar
**51**	AT2G34690[LT, LL, Ap]	0.74	Accelerated cell death 11
**52**	AT1G29970[Ap]	0.86	60S ribosomal protein L18a-like protein
**53**	*AT5G17290[Ap]	0.83	ATG5 Autophagy protein
**54**	AT1G28960[Ap]	0.76	Nudix hydrolase 15, mitochondrial
**55**	AT5G64880[Ap]	0.75	Transmembrane protein
**56**	*AT4G20380[Ap]	0.74	Protein LSD1
**57**	*AT2G07180	0.85	Probable serine/threonine-protein kinase PBL17
**58**	*AT4G26400	0.85	RING/U-box superfamily protein
**59**	AT5G53360	0.84	TRAF-like superfamily protein
**60**	*AT2G42780	0.83	Transcription elongation factor B polypeptide
**61**	*AT5G32440	0.83	Ubiquitin system component Cue protein
**62**	*AT3G07870	0.83	F-Box PROTEIN92

**63**	AT5G51400	0.82	GENE1000 PLAC8 family
**64**	AT2G46900	0.82	Transcription factor-like protein
**65**	*AT5G64920	0.82	CIP8 E3 ubiquitination
**66**	AT4G08330	0.82	Hypothetical protein
**67**	*AT4G03030	0.82	OR23 F-box/Kelch repeat family
**68**	AT5G16110	0.82	Hypothetical protein
**69**	AT2G18280	0.81	Tubby-like protein 2
**70**	AT1G15400	0.81	Hypothetical protein
**71**	AT1G15350	0.81	DUF4050 family protein
**72**	*AT1G06700	0.81	PTI1-like tyrosine-protein kinase 1
**73**	AT1G33250	0.81	Beta-1,3-n-acetylglucosaminyltransferase radical fringe protein
**74**	*AT4G27880	0.81	SINAT4 E3 ubiquitin-dependent protein catabolic process
**75**	AT5G23670	0.81	Long chain base
**76**	*AT5G16480	0.80	Plant/Fungal atypical dual-specificity phosphatase
**77**	*AT5G46150	0.80	Putative ALA-interacting subunit 2
**78**	AT5G56750	0.80	N-MYC down-regulated-like 1
**79**	AT2G47700	0.80	RFI2 RING/U-box super family
**80**	*AT2G39100	0.79	Putative RING zinc finger protein
**81**	AT2G46490	0.79	Uncharacterized protein
**82**	AT3G58670	0.79	Plant cysteine oxidase 5
**83**	AT4G15780	0.79	Vesicle-associated membrane protein 724
**84**	*AT3G61160	0.79	Shaggy-related protein kinase beta

**85**	AT1G33050	0.79	Protein DETOXIFICATION
**86**	AT1G78895	0.79	Reticulon-like protein B22
**87**	AT1G49170	0.79	Uncharacterized protein
**88**	AT3G02700	0.79	UNE6
**89**	AT1G56090	0.79	Aspartate aminotransferase
**90**	AT1G13570	0.79	F-box/FBD/LRR-repeat protein
**91**	AT1G12790	0.79	Protein PARTING DANCERS
**92**	AT5G67380	0.78	Casein kinase II subunit alpha-1
**93**	AT2G26210	0.78	Ankyrin repeat family protein
**94**	AT3G60300	0.78	RWD domain-containing protein
**95**	AT3G55770	0.78	LIM domain-containing protein WLIM2b
**96**	AT1G43700	0.78	VIP1i Transcription factor
**97**	*AT3G10770	0.78	Single-stranded nucleic acid binding R3H protein
**98**	*AT2G29400	0.78	Serine/threonine-protein phosphatase PP1 isozyme 1
**99**	AT4G12570	0.78	E3 ubiquitin-protein ligase UPL5
**100**	AT1G47270	0.78	Tubby-like F-box protein 6
**101**	AT1G73380	0.78	Uncharacterized protein
**102**	AT1G12810	0.77	Uncharacterized protein
**103**	AT5G03470	0.77	B'ALPHA Serine/threonine protein phosphatase 2A
**104**	AT5G23380	0.77	Hexosyltransferase
**105**	AT5G05750	0.77	DNAJ heat shock N-terminal domain-containing protein

**106**	AT3G02340	0.77	Uncharacterized protein
**107**	AT2G46260	0.77	BTB/POZ domain-containing protein
**108**	AT2G15570	0.76	GAT1 Thioredoxin M3, chloroplastic
**109**	AT2G15240	0.76	Uncharacterized protein
**110**	AT3G03020	0.76	WOX11
**111**	AT2G37478	0.76	CPuORF52 conserved PEPTIDE upstream reading frame
**112**	AT2G31350	0.76	Hydroxyacylglutathione hydrolase 2, mitochondrial
**113**	AT5G01800	0.76	Saposin B domain-containing protein
**114**	AT5G40190	0.76	RING-type E3 ubiquitin transferase
**115**	AT5G58575	0.76	SAGA-associated factor 11
**116**	AT4G27750	0.76	ISI1 binding protein
**117**	AT5G12390	0.76	Mitochondrial fission 1 protein B
**118**	AT5G14390	0.76	Alpha/beta-hydrolases superfamily protein
**119**	AT2G41710	0.76	AP2-like ethylene-responsive transcription factor
**120**	AT3G11530	0.75	Vacuolar protein sorting 55 family protein
**121**	AT3G05250	0.75	RING/U-box superfamily protein
**122**	AT1G51550	0.75	F-box/Kelch-repeat protein
**123**	AT5G37055	0.75	SWC6 SWR1 complex subunit 6
**124**	AT2G42890	0.75	Protein MEI2-like 2
**125**	AT5G45360	0.75	F-box protein SKIP31
**126**	AT5G45080	0.75	HGO Protein PHLOEM PROTEIN 2-LIKE A6
**127**	AT1G19680	0.75	Uncharacterized protein

**128**	AT3G01850	0.75	Ribulose-phosphate 3-epimerase
**129**	AT5G20030	0.75	NUDX19
**130**	AT2G20740	0.75	TOM2AH3 Tetraspanin-19
**131**	AT3G12630	0.75	A20 and AN1 domain-containing stress-associated protein 5
**132**	AT5G43930	0.74	Transducin family protein/WD-40 repeat family protein
**133**	AT5G04880	0.74	Laccase
**134**	AT2G44850	0.74	Uncharacterized protein
**135**	AT1G69220	0.74	Protein kinase superfamily protein
**136**	AT5G20120	0.74	Uncharacterized protein
**137**	AT5G05100	0.74	Single-stranded nucleic acid binding SH4 protein
**138**	AT1G14290	0.74	Sphinganine C4-monooxygenase 2
**139**	AT4G32760	0.74	TOM1-like protein 9
**140**	AT2G21950	0.74	F-box/Kelch-repeat protein SKIP6
**141**	AT3G22290	0.74	Endoplasmic reticulum vesicle transporter protein
**142**	AT1G14740	0.74	Protein OBERON3
**143**	AT1G20880	0.74	V-type proton ATPase proteolipid subunit
**144**	AT4G31080	0.74	Integral membrane metal-binding family protein
**145**	AT2G18670	0.74	RING-H2 finger protein ATL56
**146**	AT3G09030	0.74	BTB/POZ domain-containing protein
**147**	AT1G27290	0.74	Transmembrane protein
**148**	AT4G24370	0.74	SUPPRESSOR OF K^+ TRANSPORT GROWTH DEFECT 1

**149**	AT4G00560	0.74	NAD(P)-binding Rossmann-fold superfamily protein
**150**	*AT3G09320	0.74	Probable protein S-acyltransferase 16
**151**	AT5G41560	0.73	DET1 complexing ubiquitin ligase
**152**	AT3G12570	0.73	FYD-FYD
**153**	AT5G23590	0.73	Uncharacterized protein
**154**	AT1G26665	0.73	Mediator of RNA polymerase II transcription subunit 10b
**155**	AT5G21170	0.73	SNF1-related protein kinase regulatory subunit beta-1
**156**	AT1G70360	0.73	F-box protein
**157**	AT5G47180	0.73	Vesicle-associated protein 21
**158**	AT3G04880	0.73	DNA-damage-repair/toleration protein 102
**159**	AT2G17440	0.73	Plant intraccellular Ras-group-related LRR protein 5
**160**	At3g10770	0.73	Sequence-specific DNA binding transcription factor
**161**	AT3G62290	0.73	ADP-ribosylation factor A1E
**162**	AT5G38895	0.73	RING/U-box superfamily protein
**163**	AT5G05710	0.73	CASP-like protein
**164**	AT5G14240	0.73	Carbonic anhydrase
**165**	AT3G11770	0.73	Polynucleotidyl transferase, ribonuclease H-like family protein
**166**	AT5G63190	0.72	MA3 domain-containing protein
**167**	AT1G70150	0.72	Zinc ion binding protein
**168**	AT4G12690	0.72	DUF868 family protein
**169**	AT5G23130	0.72	Peptidoglycan-binding LysM domain-containing protein
**170**	AT5G47520	0.72	Ras-related protein RABA5a

**171**	AT1G34300	0.72	G-type lectin S-receptor-like serine/threonine-protein kinase
**172**	AT3G09880	0.72	B'BETA serine/threonine protein phosphatase 2A
**173**	AT3G18295	0.72	DUF1639 family protein, putative
**174**	AT3G07274	0.72	Pseudogene
**175**	AT4G36780	0.72	BES1/BZR1 homolog protein 2
**176**	AT1G28410	0.72	Myosin heavy chain-like protein
**177**	AT1G36070	0.72	Transducin/WD40 repeat-like superfamily protein
**178**	AT1G05090	0.72	Dentin Sialophosphoprotein-like protein
**179**	AT5G06560	0.72	Myosin-binding protein 7
**180**	AT2G21240	0.72	Protein BASIC PENTACYSTEINE 4
**181**	AT1G22890	0.72	Transmembrane protein
**182**	AT5G59380	0.72	Methyl-CpG-binding domain-containing protein 6
**183**	AT5G19950	0.72	Tudor domain protein (DUF1767)
**184**	AT3G13550	0.72	Constitutive photomorphogenesis protein 10
**185**	AT5G65960	0.72	GTP binding protein
**186**	AT4G32160	0.72	PX domain-containing protein EREL1
**187**	AT3G04350	0.72	Zeta-carotene desaturase
**188**	AT1G43130	0.71	Protein-LIKE COV 2
**189**	AT2G44900	0.71	FBX5 protein ARABIDILLO 1
**190**	AT5G22360	0.71	Vesicle-associated membrane protein 714
**191**	AT1G34150	0.71	tRNA pseudouridine synthase

**192**	AT4G31250	0.71	Probable LRR receptor-like serine/threonine-protein kinase
**193**	AT1G60140	0.71	Trehalose phosphate synthase 10
**194**	AT4G39910	0.71	Ubiquitin carboxyl-terminal hydrolase 3
**195**	AT1G09280	0.71	Rhodanese-like domain-containing protein 6
**196**	AT4G11240	0.71	Serine/threonine-protein phosphatase PP1 isozyme 6
**197**	AT4G38260	0.71	Transport/Golgi organization-like protein
**198**	AT1G67710	0.71	ARR11 Two-component response regulator
**199**	AT4G24990	0.71	Membrane-anchored ubiquitin-fold protein 3
**200**	AT2G31810	0.71	Acetolactate synthase small subunit 2, chloroplastic

PT: Peroxisomal transport (P = $3.05e^{-08}$), IPTI: Intracellular protein transmembrane import (P = $2.93e^{-07}$), PO: Peroxisome organization (P = $3.05e^{-08}$), PF: Photoperiodic flowering (P = $1.85e^{-15}$), EPLP: Establishment of protein localization to peroxisome (P = $3.05e^{-08}$), PLP: Protein localization to peroxisome (P = $3.05e^{-08}$), LM: Lipid modification (P = $3.22e^{-02}$), FABO: Fatty acid beta-oxidation (P = $3.79e^{-03}$), PTP: Protein targeting to peroxisome (P = $3.05e^{-08}$), MACP: Monocarboxylic acid catabolic process (P = $1.88e^{-02}$), IPTT: Intracellular protein transmembrane transport (P = $3.82e^{-07}$), LO: Lipid oxidation (P = $4.68e^{-03}$), PI: Protein import (P = $2.76e^{-04}$), FAO: Fatty acid oxidation (P = $4.00e^{-03}$), FACP: Fatty acid catabolic process (P = $1.55e^{-02}$), I-GV-MT: Intra-Golgi vesicle-mediated transport (P = $2.52e^{-07}$), LT: Lipid transport (P = $1.02e^{-03}$), VMT: Vesicle mediated transport (P = 8.4^{-03}), LL: Lipid localization (P = $2.76e^{-03}$), PTT: Protein transmembrane transport (P = $3.82e^{-07}$), CLCP: Cellular lipid catabolic process (P = $2.92e^{-02}$), PIPM: Protein import into peroxisome matrix (P = $3.05e^{-08}$), LIT: Lithium ion transport (P = $5.38e^{-06}$), PP: Photoperiodism (P = $2.79e^{-15}$), VRTM: Vegetative to reproductive phase transition of meristem (P = $8.71e^{-11}$), PLV: Protein localization to vacuole (P = $3.32e^{-02}$), EPLV: Establishment of protein localization to vacuole (P = $3.32e^{-02}$), PTV: Protein targeting to vacuole (P = $3.32e^{-02}$), SB: Snare binding (P = $2.03e^{-06}$), SRA: SNAP receptor activity (P = $1.84e^{-06}$), mRNACP: mRNA catabolic process (P = $2.32e^{-02}$), RNACP: RNA catabolic process (P = $3.29e^{-02}$), N-TMCP: Nuclear-transcribed mRNA catabolic process (P = $2.22e^{-02}$), RB: Response to brassinosteroid (P = $2.04e^{-02}$), Ap: Autophagy (P = $1.21e^{-03}$). *25 expression-correlated genes selected for stimulus-specific microarray analysis.

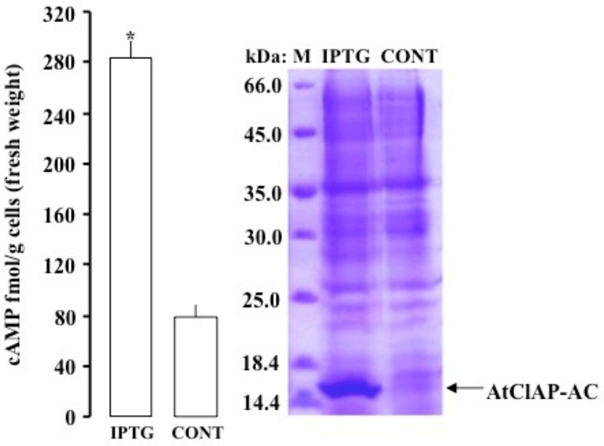

Figure S1. Cyclic AMP generated by the induced (IPTG) and un-induced (CONT) *E. cloni* EXPRESS BL21 (DE3) pLysS DUOs cells harbouring the AtClAP-AC gene fragment. The cAMP was measured by enzyme immunoassay following the acetylation protocol described in the supplier's manual (Sigma-Aldrich Corporation, code: CA201). Inset: SDS-PAGE of protein fractions from the induced (IPTG) and un-induced (CONT) cell cultures. Data are mean values (n = 3) and error bars show SE of the mean. Asterisk indicates significant difference ($p < 0.05$) determined by ANOVA and *post hoc* Student–Newman–Keuls multiple range tests.

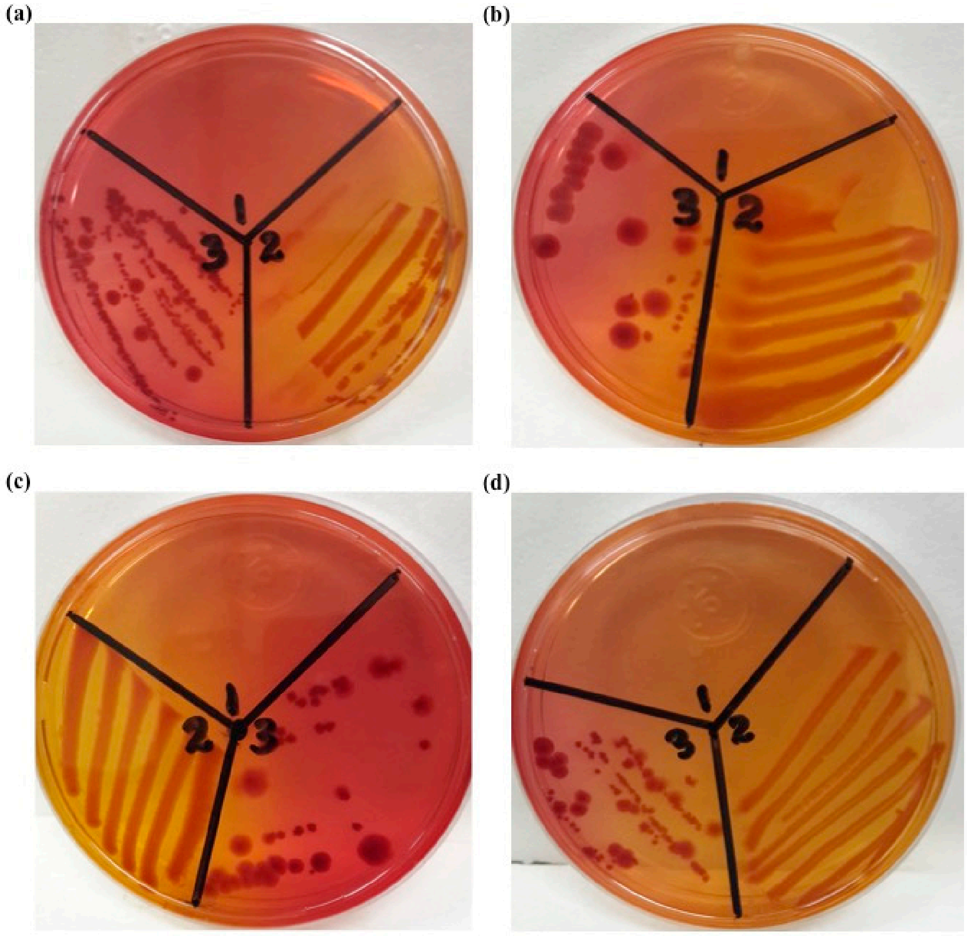

Figure S2. (**a**–**d**) Crystal violet containing MacConkey agar plates supplemented with 15 µg/L kanamycin and 0.1 mM IPTG, and uniformly inoculated with (**1**) no cells, (**2**) *cya*A mutant cells and (**3**) *cya*A mutant cells transformed with the pTrcHis2-TOPO:AtClAP-AC fusion construct. The plates were incubated at 37°C for 40 h to develop the displayed colony growth patterns.
